# Racial Disparities in Route of Hysterectomy for Benign Indications Within an Integrated Health Care System

**DOI:** 10.1001/jamanetworkopen.2019.17004

**Published:** 2019-12-06

**Authors:** Eve Zaritsky, Anthonia Ojo, Lue-Yen Tucker, Tina R. Raine-Bennett

**Affiliations:** 1 Obstetrics and Gynecology, Kaiser Permanente Northern California, Oakland Medical Center, Oakland; 2 Division of Research, Kaiser Permanente Northern California, Oakland; 3 Division of Research, Obstetrics and Gynecology, Kaiser Permanente Northern California, Oakland Medical Center, Oakland

## Abstract

This cross-sectional study examines racial disparities in the route of hysterectomy for benign indications within an integrated health care system in the United States.

## Introduction

Disparities exist in benign gynecological surgery within the United States; racial/ethnic minority groups are less likely to receive minimally invasive hysterectomies (MIHs).^1^ Compared with open abdominal hysterectomies, MIHs (including laparoscopic, vaginal, and robotic procedures) provide benefits such as reduction in postoperative pain, blood loss, and recovery time.^2^ The study by Ranjit et al^1^ demonstrated that white patients received MIH at higher rates even within a universal insurance system. In this study, we investigated whether racial disparities would be significantly reduced or eliminated within the context of an integrated health care system, Kaiser Permanente Northern California (KPNC).

## Methods

This single-institution cross-sectional study included patients aged 18 years and older undergoing hysterectomy for benign indications at KPNC hospitals from January 1, 2008, to December 31, 2015. Data collection and validation methods have been described previously.^3,4^ During this period, KPNC underwent a 4-pronged quality improvement initiative involving leadership engagement, surgeon training, reduction of low-volume surgeons, and encouragement of best practices to increase the MIH rate.^3,4^ Patient and clinical characteristics associated with MIH were assessed using multivariable logistic regression. Poisson regression models tested for linear trends of MIH by race/ethnicity controlling for age, body mass index, median household income, parity, comorbidity index score, uterine weight, surgical indication, concomitant procedures, surgeon hysterectomy volume, and a linear term for year of hysterectomy. Race/ethnicity was self-reported by patients. These secondary analyses were performed in 2019 using SAS software version 9.4 (SAS Institute). The threshold for statistical significance was set at 2-sided *P* < .05. Strengthening the Reporting of Observational Studies in Epidemiology (STROBE) reporting guidelines were followed. The KPNC institutional review board approved this study with waiver of informed consent because it was conducted using only deidentified patient data.

## Results

Among the 31 385 patients who underwent hysterectomies in KPNC facilities, 15 384 (49%) were white, 4095 (13%) were African American, 6721 (21.4%) were Hispanic, 3599 (11.5%) were Asian, and 1586 (5.1%) were another race/ethnicity. In all, 4847 patients (15.4%) were aged 18 to 39 years; 15 514 (49.4%), 40 to 49 years; 6902 (21.9%), 50 to 59 years; and 4122 (13.1%), older than 59 years. A total of 22 865 hysterectomies (72.9%) were MIH, including 15 086 (66.0%) laparoscopic (979 of which were robotic) and 7779 (34.0%) vaginal. Overall, the discrepancy in proportions of MIH between African American and white patients decreased over time (Figure). In early 2008, at the beginning of the MIH initiative, racial/ethnic minority patients were less likely to receive MIH than white patients (adjusted odds ratio [aOR], 0.64; 95% CI, 0.49-0.83). By 2010, MIH rate was no longer associated with race. In 2015, Asian patients were significantly more likely to receive MIH than white patients (aOR, 1.79; 95% CI, 1.02-3.20) (Table). Linear trend test showed that MIH increased at an annual relative rate of 1.098 (95% CI, 1.093-1.103; *P* < .001) from 2008 to 2015. Specifically, MIH increased at an annual relative rate of 1.147 for African American patients (95% CI, 1.135-1.161; *P* < .001), 1.122 for Asian patients (95% CI, 1.109-1.135; *P* < .001), 1.101 for patients of other race (95% CI, 1.084-1.119; *P* < .001), 1.088 for Hispanic patients (95% CI, 1.079-1.097; *P* < .001), and 1.088 for white patients (95% CI, 1.082-1.094; *P* < .001). Minimally invasive hysterectomy was also significantly associated with all other factors included in the model except for income. The proportion of MIHs performed by high-volume surgeons increased steadily, while the proportion performed by low-volume surgeons decreased from 70% to 30%; the surgeon pool decreased voluntarily from 416 to 234 surgeons.^4^

**Figure.**
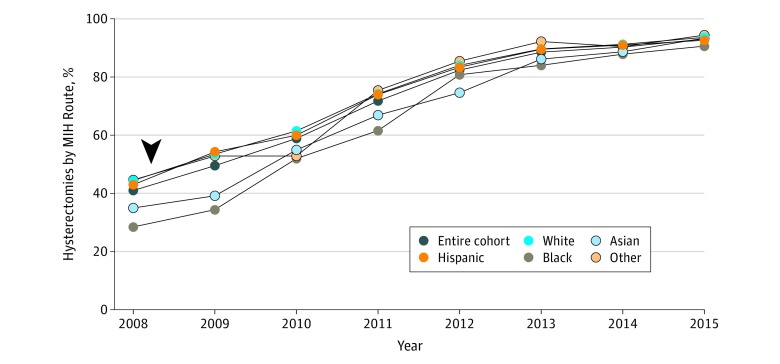
Unadjusted Proportions of Minimally Invasive Hysterectomies (MIHs) Performed for Benign Gynecological Conditions by Racial/Ethnic Group, 2008-2015, Kaiser Permanente Northern California The arrowhead denotes the beginning of the quality improvement intervention in early 2008.

**Table.** Adjusted Odds Ratios of Receiving a Minimally Invasive Hysterectomy by Race/Ethnicity^a^

Race/Ethnicity	Adjusted Odds Ratio (95% CI)							
	2008	2009	2010	2011	2012	2013	2014	2015
White	1 [Reference]	1 [Reference]	1 [Reference]	1 [Reference]	1 [Reference]	1 [Reference]	1 [Reference]	1 [Reference]
Hispanic	0.84 (0.67-1.04)	0.74 (0.58-0.95)^b^	0.93 (0.76-1.31)	0.96 (0.77-1.20)	0.82 (0.63-1.07)	0.85 (0.62-1.16)	0.81 (0.57-1.16)	1.28 (0.85-1.97)
African American	0.85 (0.65-1.10)	0.64 (0.49-0.83)^b^	0.84 (0.74-1.19)	0.85 (0.65-1.12)	1.11 (0.82-1.52)	0.73 (0.51-1.06)	0.88 (0.57-1.37)	1.29 (0.75-2.26)
Asian, Pacific Islander, or Hawaiian	0.75 (0.57-0.99)^b^	0.64 (0.49-0.83)^b^	0.88 (0.68-1.14)	0.91 (0.69-1.20)	0.67 (0.50-0.92)^b^	0.96 (0.67-1.37)	0.91 (0.59-1.42)	1.79 (1.02-3.20)^b^
Other	0.97 (0.68-1.38)	1.0 (0.71-1.41)	0.76 (0.54-1.07)	1.20 (0.80-1.82)	1.08 (0.69-1.74)	1.45 (0.83-2.66)	1.17 (0.64-2.21)	1.48 (0.67-3.57)

^a^ Data were estimated using logistic regression controlling for age, body mass index, median household income, parity, comorbidity index score, uterine weight, surgical indication, concomitant procedures, and surgeon hysterectomy volume.

^b^ Statistically significant at *P* < .05.

## Discussion

This study found that racial disparities in MIH no longer persisted within this integrated health care system, unlike in other settings within the United States.^1,5,6^ The study by Alexander et al^5^ found that African American individuals were approximately twice as likely to undergo open abdominal hysterectomies vs MIHs compared with their white counterparts. The study by Pollack et al^6^ demonstrated that despite increasing annual laparoscopic rates, racial/ethnic minority women were less likely to undergo MIH. Our study found a significant increase in MIH, with a higher annual relative rate increase in MIH for racial/ethnic minority patients than for their white counterparts. Limitations of this study include the inability to identify determinants of racial disparities, undercapture of previous pelvic surgery, and lack of generalizability to other practice models. Our results may be due to system changes.^4,5^ Our initiative increased MIH rates and the proportion of high-volume surgeons while simultaneously reducing the surgeon pool. With these system changes, we observed a reduction of racial disparities in MIH.
